# Correction to: The quintessence of metallomics: a harbinger of a different life science based on the periodic table of the bioelements

**DOI:** 10.1093/mtomcs/mfac071

**Published:** 2023-02-23

This is a correction to: Wolfgang Maret, The quintessence of metallomics: a harbinger of a different life science based on the periodic table of the bioelements, *Metallomics*, Volume 14, Issue 8, August 2022, mfac051, https://doi.org/10.1093/mtomcs/mfac051

In the originally published version of this manuscript, the credit line under Fig. 1 incorrectly stated that ‘Figure was adopted from W. Maret, Metallomics: The Science of Biometals and Biometalloids, chapter 1 in Metallomics, M. A. Z. Arruda, ed, Advances in Experimental Medicine and Biology 1055, 1–20, 2018 with permission of Springer International Publishing AG.’

The correct version of the credit line under Fig. 1 is: ‘Figure was adapted from W. Maret, Metallomics: The Science of Biometals and Biometalloids, chapter 1 in Metallomics, M. A. Z. Arruda, ed, Advances in Experimental Medicine and Biology 1055, 1–20, 2018 with permission of Springer International Publishing AG.’

There was an error in the sentence: “The amounts vary over at least three orders of magnitude from 18 mg iron to 2.4 µg vitamin B12 (cobalt).”

The correct version of this sentence is: “The amounts vary over at least three orders of magnitude from 18 mg iron for adult females to 2.4 µg vitamin B12 (cobalt) recommended per day.”

Table 3 incorrectly referred to 1 : 1 : 1. It should refer to 1 : 1 instead.

These errors have been corrected online.

